# Improved method of magnification factor calculation for the angiographic measurement of neurovascular lesion dimensions

**DOI:** 10.1120/jacmp.v3i3.2573

**Published:** 2002-06-01

**Authors:** Zhou Wang, Stephen Rudin, Daniel R. Bednarek, Laszlo Miskolczi

**Affiliations:** ^1^ Department of Physiology and Biophysics State University of New York at Buffalo 3435 Main Street Buffalo New York 14214; ^2^ Department of Radiology State University of New York at Buffalo 3435 Main Street Buffalo New York 14214; ^3^ Department of Physics State University of New York at Buffalo Buffalo New York 14260; ^4^ Department of Neurosurgery State University of New York at Buffalo 3435 Main Street Buffalo New York 14214; ^5^ Erie County Medical Center 462 Grider Street Buffalo New York 14215; ^6^ Toshiba Stroke Research Center State University of New York at Buffalo 3435 Main Street Buffalo New York 14214

**Keywords:** angiography, neurovascular lesion, magnification, calibration, size measurement

## Abstract

Accurately evaluating the size of a neurovascular lesion is essential for properly devising treatment strategies. The magnification factor must be considered in order to measure the dimension of a lesion from an angiogram. Although a method to calculate the magnification of the lesion by linear interpolation of the measurable magnification factors of two markers has been in use, this paper shows that it can be inaccurate. By deriving the exact formula for calculating the magnification factor at the level of the lesion, the error generated by the linear interpolation of magnification factor has been evaluated. This error was found to depend on source‐to‐skin distance (SSD), the location of the lesion in the head, and the head size. The closer the head is to the focal spot and the nearer the lesion is to the center of the head, the larger is the error. Since clinicians tend to use high geometric magnification (i.e., small SSD) in interventional procedures, there exists a possible consequential error of more than 3% in lesion sizing if the linear‐interpolation calculation method is used. It is thus recommended that the exact formula derived here be used to calculate the magnification factor to improve accuracy.

PACS number(s): 87.57.–s, 87.57.Nk, 87.59.Dj

## INTRODUCTION

**Figure 1:**
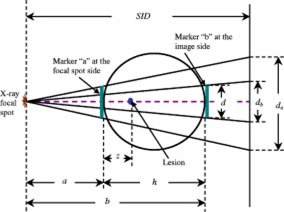
(Color) Diagram of two‐marker method for measuring the magnification factor at the location of the lesion. The lateral view of a PA projection is shown.

Accurately evaluating the sizes of neurovascular lesions such as aneurysms, stenoses, and arteriovenous malformations is essential for properly devising treatment strategies. The commonly used technique is to measure the lesion size in an angiogram and scale it with a factor for geometric magnification. There are three methods in use for estimating magnification factor in an angiogram. One method is to place a circular marker of known size on the surface of the patient's head and calculate the magnification factor from the image of the marker. This is obviously inaccurate because the magnification factor at different depths in the head is not the same as at the surface. In a second method, the outside diameter of a catheter placed at or near the plane of the vascular lesion can be used as a reference. However, accuracy of measuring the catheter diameter in an angiogram is limited by the resolution of the imaging system1–3 and the x‐ray attenuation of the catheter wall.^4^ In a third method,^5^ two markers are placed on opposite sides of the head and the magnification factor at the level of the lesion is calculated by linear interpolation of the two magnification factors of the markers (Fig. 1). This method is shown in this study to provide inaccurate results, because the true magnification factor does not vary in a linear fashion with depth. We derive the formula for the exact interpolation of the magnification factor in the two‐marker method. The improvement in accuracy achieved by using this exact interpolation method was evaluated by calculating the magnitude of measurement errors introduced by linear interpolation of the magnification factor.

## METHODS

The measurement procedure for the two‐marker method involves placing two radio‐opaque markers of known size *d* on opposite sides of the head is shown in Fig. 1. By taking a lateral view of the head, the relative location of the lesion between the two markers is measured as *R*, where R=z/h (*z* and *h* are defined as shown in Fig. 1). If the size of marker “*a*” measured in the posterior‐anterior (PA) image plane is $d_{a}$ and the size of marker “*b*” measured in the PA image plane is $d_{b}$ (Fig. 1), the magnification factors at location “*a*” and “*b*” can be calculated as: $M_{a}=d_{a}/d$ and $M_{b}=d_{b}/d$.

**Figure 2:**
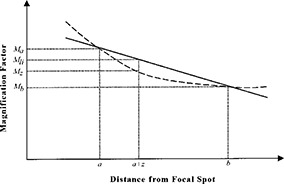
Linear interpolation of the magnification factor (——) and the actual magnification factor (—‐).

The currently used linear interpolation method5 is shown in Fig. 2 by the straight line connecting the calculated marker magnification factors at “*a*” and “*b*”. The magnification factor of the lesion is calculated by the linear interpolation of the magnification factors for the two markers as

(1) $$M_{li}=(1-R)\cdot M_{a}+R\cdot M_{b},$$

where

(1.1) $$R=\frac{z}{h}$$

and

(1.2) h=b−a

as measured on the lateral image.

In fact, the relationship between magnification and location is nonlinear (as shown by the dashed line in Fig. 2). Therefore, we need to derive the exact formula to calculate the magnification of the lesion. From Fig. 1, we can calculate the magnification at the depth of the lesion as

(2) $$M_{z}=\frac{\text{SID}}{a+z},$$

where SID is the source‐to‐image distance shown in Fig. 1

(2.1) $$\text{SID}=aM_{a}=bM_{b},$$

By substitution of Eq. (1.1), (1.2), and (2.1) into Eq. (2), we can obtain the magnification factor at the level of the lesion as:

(3) $$M_{z}=\frac{M_{a}M_{b}}{M_{b}(1-R)+M_{a}R}.$$

This is equivalent to performing a linear interpolation of the inverse magnification:

(4) $$\frac{1}{M_{z}}=(1-R)\frac{1}{M_{a}}+R\frac{1}{M_{b}}.$$

Therefore, the percentage error introduced by linear interpolation of the magnification factor is given by:

(5) $$\text{Percentage}\;\text{Error}=100\times \frac{M_{li}-M_{z}}{M_{z}}.$$

## RESULTS AND DISCUSSION

**Figure 3:**
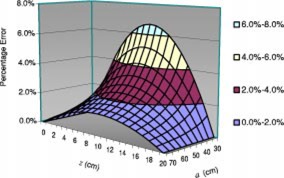
(Color) Error caused by the linear interpolation of the magnification method as a function of source‐to‐skin distance (SSD), a, and the depth of lesion, *z* (SID=100 cm and h=20 cm).

Errors generated by linearly interpolating the magnification factor are shown in Fig. 3 for a fixed SID (100 cm) and head size (20 cm). As shown in Fig. 3, the closer the head is to the focal spot (smaller *a*) and the nearer the lesion is to the center of the head (at z=10 cm), the larger is the error. We can see that the error is 3% when the source to skin distance, a, is 50 cm for a geometric magnification factor of 2, and the maximum error increases to 6.7% when the head is 30 cm from the focal spot (a=30 cm).

**Figure 4:**
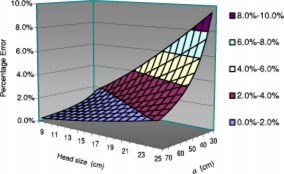
(Color) Error caused by the linear interpolation of the magnification method as a function of SSD, *a*, and head size, *h* (SID=100 cm, lesion is located at the center of the head).

Figure 4 illustrates the relation between the head size and the error caused by linear interpolation of the magnification factor. It can be seen that larger head size may cause larger errors, resulting in a maximum error of 9.5% when the head size is 25 cm. In addition, Fig. 4 shows that the percentage errors increase with smaller source‐to‐skin distance (SSD) for a given head size.

Since clinicians tend to use high geometric magnification (i.e., small SSD) during interventional neurovascular procedures, the linear‐interpolation calculation method can introduce a possible consequential error of more than 3% in lesion sizing when a geometric magnification of 2 is used with an SSD of 50 cm and more than 6% when the head is placed even closer to the source. When feature sizing, this error in magnification factor determination is in addition to the error in lesion or vessel boundary estimation,2,3 which can by itself add an uncertainty of 0.1 mm or 3% to 10% for vessel or stenosis dimensions of 3 to 1 mm, respectively. Such errors can cause failure of an intervention when, for example, a stent is allowed to migrate due to undersizing.

All of these errors become more significant when quantitative flow determinations must be made since then an area or volume determination propagates the linear errors discussed here. For example, a nonrandom 3% error in magnification factor determination will propagate to greater than 9% error and a 6% magnification factor error will propagate to greater than 19% error when used to determine blood volume since the error is multiplicative.

## CONCLUSION

Direct linear interpolation of magnification factors using the two‐marker method can cause errors in the estimation of lesion dimensions in angiograms. In order to calculate neurovascular lesion dimensions more accurately, the authors recommend a method that uses linear interpolation of the inverse magnification, which is in fact the exact magnification calculation method based on using two markers. The same measurements are made with both methods and the calculation is no more complicated to perform using the exact inverse magnification equation than that for linear interpolation.

## ACKNOWLEDGMENT

This work was supported in part by NIH Grant No. IR01NS38745, an equipment grant from the Toshiba Corp. and a grant from the John R. Oishei Foundation.
